# Pea Within Pea: Microencapsulation of Pea Pod Extract Using Pea Grain Powder as a Sustainable Carrier

**DOI:** 10.3390/plants15070996

**Published:** 2026-03-24

**Authors:** Nada Ćujić Nikolić, Zorana Mutavski, Jelena Mudrić, Milica Radan, Jelena Vulić, Smilja Marković, Katarina Šavikin

**Affiliations:** 1 Institute for Medicinal Plants Research “Dr. Josif Pančić”, Tadeuša Košćuška 1, 11000 Belgrade, Serbia; ncujic@mocbilja.rs (N.Ć.N.); jmudric@mocbilja.rs (J.M.); mradan@mocbilja.rs (M.R.); ksavikin@mocbilja.rs (K.Š.); 2 Faculty of Technology, University of Novi Sad, Bulevar Cara Lazara 1, 21000 Novi Sad, Serbia; jvulic@uns.ac.rs; 3 Institute of Technical Sciences of Serbian Academy of Sciences and Arts, Knez Mihailova 35/IV, 11000 Belgrade, Serbia; smilja.markovic@itn.sanu.ac.rs

**Keywords:** *Pisum sativum* L., circular bio economy, ultrasound-assisted extraction, microencapsulation, pea pod valorization, pea-based protein carrier, natural pigments

## Abstract

The pods of pea (*Pisum sativum* L.), an abundant agroindustry by-product, represents a sustainable source of bioactive compounds. To harness these compounds effectively, this study aimed to optimize the ultrasound-assisted extraction (UAE) of polyphenols and plant pigments (chlorophylls and carotenoids) from pea pod waste using response surface methodology, and to evaluate the encapsulation of the resulting extract with a novel pea-based carrier derived from whole pea grain powder. The optimal conditions for the extraction were a time of 45 min, a solid-to-solvent ratio of 1:48 (*w*/*v*), and an ethanol concentration of 58.51% (*v*/*v*). The extract obtained under these conditions was encapsulated using pea grain powder and compared with a conventional whey protein carrier. The resulting microencapsulates were characterized in terms of process yield, moisture content, particle size distribution, thermal properties, and phenolic composition. Pea grain powder as a carrier provided higher powder yield, lower moisture content, and improved thermal stability, whereas whey protein allowed slightly higher retention of most bioactive compounds, except for coumaric acid and kaempferol. Overall, these findings highlight pea grain powder as a promising plant-based carrier that supports the valorization of pea pod waste, contributing to the development of sustainable ingredients and a circular economy for legume processing by-products.

## 1. Introduction

The increasing demand for sustainable food systems, as well as the rapid expansion of the plant-based market, have intensified research on the valorization of agro-industrial waste as a valuable source of bioactive compounds and functional ingredients [1,2,3,4]. Among leguminous crops, pea (*Pisum sativum* L.) occupies an important place due to its high protein content, good amino acid profile, and broad applicability in the food and pharmaceutical industries [1,5]. However, pea processing generates substantial amounts of by-products, particularly pea pods, which are often discarded as waste or used as low-value animal feed despite being rich in bioactive and nutritional compounds [6]. The valorization of pea pod residues thus represents an opportunity for waste reduction and the creation of new value, contributing to the circular bio-economy [7].

Pea pods are still underutilized but a promising source of bioactive molecules such as polyphenols, flavonoids, and plant pigments, including chlorophylls *a* and *b*, and carotenoids [8,9]. These compounds exhibit significant antioxidant, anti-inflammatory, and antimicrobial activities that could be significant for the development of new functional foods and nutraceuticals [10]. On the other hand, their incorporation into formulations is often difficult because of their poor stability, low solubility, and susceptibility to degradation upon exposure to light, oxygen, and elevated temperature [11,12]. Therefore, the protection and controlled delivery of these molecules through encapsulation systems are methods to preserve their bioactivity and ensure their bioavailability in target applications [13,14].

Microencapsulation is considered an efficient technology process for stabilizing and delivering sensitive bioactives, and the choice of carrier material is critical in determining encapsulation efficiency and physicochemical stability [15]. While whey protein is widely used because of its emulsifying and film-forming abilities, the growing demand for allergen-free, plant-based alternatives has brought into focus protein carriers derived from sustainable plant sources [6]. In this context, pea protein and pea grain powder are particularly attractive due to their nutritional value and favorable technological properties (proper taste, solubility, emulsification, gelation) [16,17].

Incorporating pea pod extracts into a pea-based carrier system provides a unique “pea within pea” valorization concept, coupling bioactive-rich pea pod waste with a pea-derived carrier for encapsulation. Our approach thus promotes complete utilization of a single crop. Moreover, synergistic interactions between pea polyphenols and pea proteins may further improve encapsulation performance, oxidative stability, and controlled release behavior [18,19].

Accordingly, the present study aims to develop a sustainable and innovative microencapsulation system by valorizing pea pod waste as a source of natural bioactives and employing pea grain powder as a biocompatible carrier. The work involves the optimization of extraction procedures for the recovery of polyphenols and pigments from pea pods (with the principle goal of being economically justified and environmentally friendly, based on the principles of “green extraction”), encapsulation of the obtained extract in a pea carrier, detailed physicochemical characterization of the resulting encapsulates (FTIR, DSC, particle size distribution), and comparison with a conventional whey protein-based carrier. This combined use of pea by-products and whole grains represents a novel, minimally processed approach that aligns with circular economy principles by transforming legume processing residues into functional ingredients for food and pharmaceutical applications.

## 2. Materials and Methods

### 2.1. Pea Pod Plant Material Preparation

Pea seeds and pea pod samples were collected across Serbia during the last two weeks of June 2025. The whole peas were harvested in the yard of the village of Putinci, near the town of Ruma, in Serbia. Then, the pea pods were manually separated from the grains. The pea pods were air-dried (in the shade of the sun, away from direct sunlight) at room temperature for seven days. The dried sample of pea pods was ground into a fine powder in a laboratory mill, then separated using a sieve with a 0.75 mm mesh (Yugoslavian Pharmacopeia). After sieving, the dried sample fraction with a particle size diameter between 0.30 and 0.75 mm (300 and 750 µm) was selected and used for further analyses; this size range classifies the powder as moderately fine. Dried residues of pea pod biomass were determined on a moisture determination device (Halogen Moisture Analyzer HB43-S, Mettler Toledo, Greifensee, Switzerland) and then subjected to an extraction process. The determined moisture content of pea pod biomass was 5.28%.

### 2.2. Preparation of Pea Grain Powder (Pea-Based Carrier)

In parallel to pea pod material preparation, pea grain powder was prepared as a future carrier using the freeze-drying process. The whole grains were frozen at −80 °C and stored in a deep freezer, then subjected to freeze-drying. The process was performed in a freeze-dryer (Beta 1–8 Freeze Dryer, Martin Christ, GmbH, Osteroide am Harz, Germany), at −60 °C, with pressures of 0.011 mbar for 24 h and 0.0012 mbar for 1 h to remove residual water further. The freeze-dried whole grains (FP) were ground into a powder using a laboratory mill and then prepared for the encapsulation process.

### 2.3. Optimization of Pea Pod Extraction Process

The extraction procedure (UAE, ultrasound-assisted extraction) was carried out in an ultrasonic water bath (Bandelin Sonorex, Berlin, Germany) with a constant frequency (35 kHz) and power (320 W), as well as under constant temperature conditions (50 °C), to minimize thermal degradation of heat-sensitive biocompounds, especially natural pigments. The total volume of the extraction mixture was 25 mL. To optimize pea pod extraction by using response surface methodology, the extracts were prepared according to the Box–Behnken design (BBD) by varying the time (15 min, 30 min, 45 min), ethanol concentration (30%, 50%, 70%), and solid-to-solvent ratios (1:10, 1:30, 1:50) at three levels. The design consisted of 15 randomized runs with three replicates at the central point (Table 1). Immediately after the extraction procedure, the extracts were filtered through filter paper into glass flasks, and the resulting samples were stored in a dark place at 4 °C until analysis. Total polyphenols, chlorophyll A, and carotenoid content were selected as responses and monitored spectrophotometrically (Section 2.6.2).

The quadratic (polynomial) model proposed for each response was

(1) $$Y_{k}=b_{k0}+\sum_{i=1}^{3}b_{ki}X_{i}+\sum_{i=1}^{3}b_{kii}X_{i}^{2}+\sum_{i=1}^{2}\sum_{j=i+1}^{3}b_{kij}X_{i}X_{j}$$

where Y_k_ is the predicted response of target compounds, b_k0_ is the intercept, b_ki_ is the linear coefficient, b_kii_ is the quadratic coefficient, b_kij_ is the interaction coefficient of variables, and X is the coded independent variable.

The influence of the ethanol concentration, time, and solid-to-solvent ratio and their interactions on the extraction efficiency of total polyphenols, chlorophyll A, and carotenoids were evaluated using analysis of variance (ANOVA), with statistical significance defined at *p* < 0.05. Regression coefficients were determined through multiple regression analysis, which enabled the construction of the final predictive models. Model adequacy was verified using several statistical criteria, including the lack-of-fit test, coefficient of determination (R^2^), adjusted R^2^, and model significance. Experimental design generation, statistical evaluation, responses visualization, and the identification of optimal extraction conditions were conducted using Design-Expert software (version 11, Stat-Ease Inc., Minneapolis, MN, USA).

### 2.4. Preparation of Optimized Pea Pod Extract

A higher amount of pea pod extract (optimal PPE) was produced using the UAE method under conditions chosen after the optimization of the extraction process. After the extraction process, ethanol was subsequently removed by a rotary evaporator with a vacuum (Buchi rotavapor R-114, Flawil, Switzerland) at 50 °C. After the evaporation process, a dealcoholized extract was used for the encapsulation process.

### 2.5. Encapsulation Process

The encapsulation technique was used for the preservation of pea pod biocompounds. After producing a larger quantity of extract, the dealcoholized liquid extract was encapsulated using the lyophilization technique (freeze drying), under the same process conditions described in Section 2.2, with and without the addition of a carrier. The concentration of carriers was 50% *w*/*w* relative to the dry matter of the extract. The optimal and dealcoholized pea pod extract was subjected to the encapsulation processes and was concentrated and encapsulated into three forms: (1) PPE, the pure pea pod extract; (2) PPE + FP, combined with freeze-dried pea grain powder; and (3) PPE + WP, combined with whey protein, as a conventional protein-based carrier. The carriers were separately dissolved in the extract and held at room temperature, for 2 h, then freeze-dried. The extracts were frozen at −80 °C and subjected to freeze-drying at −60 °C under 0.011 mbar for 24 h, followed by 0.0012 mbar for 1 h to remove residual moisture. By this strategy, encapsulates were prepared using both conventional and innovative carriers. Powders were stored in the dark, in a desiccator at room temperature, and these conditions ensured physical stability and active compound preservation.

### 2.6. Encapsulates Characterization

Powders were characterized based on technological properties (powder yield, moisture content), along with physicochemical properties (particle size distribution, thermal stability, and the FTIR analysis) and chemical composition (the analysis of total and individual compounds).

#### 2.6.1. Technological Examinations

Examinations of technological parameters, process yield, and moisture content of encapsulated powders were explained in detail in previous studies [20].

#### 2.6.2. Chemical Characterization

Total polyphenolics (TPC) and natural pigments (chlorophyll A, chlorophyll B, and carotenoids) were analyzed spectrophotometrically. All mentioned analyses are described in detail in the study by Ćujić Nikolić et al. (2025) [12]. For total polyphenolic content, the results were expressed as milligrams of gallic acid equivalent per gram of dried sample for pea pod extracts (mg GAE/g DW) and milligrams of gallic acid equivalent per gram for encapsulated powders (mg GAE/g). The results of the natural pigments (chlorophyll A, chlorophyll B, carotenoids) were expressed as micrograms of chlorophyll A and carotenoids per gram of dried pea pod sample (µg/g DW), as well as milligrams of chlorophyll A, B, and carotenoid equivalent per g of encapsulated powders (µg/g).

The chemical analysis of the individual phenolic compounds (gallic acid, epicatechin, catechin, coumaric acid, ferulic acid, sinapinic acid, kaempferol) in the obtained encapsulates was examined in detail using HPLC (High-Performance Liquid Chromatography) performed on Shimadzu Prominence equipment (Shimadzu, Kyoto, Japan), which contains an LC-20AT binary pump, CTO-20A thermostat, and SIL-20A automatic dispenser connected to a DAD detector. The separation was performed on a Luna C-18 RP column, 5 µm and 250 × 4.6 mm (Phenomenex, Torrance, CA, USA), which was protected by a C18 precolumn, 4 × 30 mm (Phenomenex, Torrance, CA, USA). The A (acetonitrile) and B (1% formic acid) were used as mobile phases, with a flow rate of 1 mL/min and a gradient profile going from 10% to 25% A from 0 to 10 min; a linear increase to 60% A from 10 to 20 min; and a further linear increase to 70% A from 20 to 30 min, followed by a return to the initial 10% A over 10 min, with an additional 5 min for equilibration. All the analyses were run in triplicate, and data acquisition was carried out by LC Solution Software version 1.25 (Shimadzu, Kyoto, Japan). The identified results were validated using reference standards, which were dissolved in 50% methanol. All samples and solvents were filtered before analysis through 0.45 µm pore size membrane filters (Millipore, Bedford, MA, USA).

#### 2.6.3. Physicochemical Characterization

The structural characterization of the examined encapsulates and their respective carriers was performed using FTIR (Fourier Transform Infrared spectroscopy, Cary 630 FTIR spectrometer, Agilent, Santa Clara, CA, USA). The particle size distribution of the obtained encapsulates was measured using a Mastersizer (Malvern, Worcestershire, UK). The thermal stability of the produced encapsulates was examined using the DSC (Differential Scanning Calorimetry) method with a DSC 131 Evo (SETARAM Instrumentation, Caluire-et-Cuire, France). All mentioned analyses are described in detail in Ćujić Nikolić et al. (2024) [14].

### 2.7. Statistics

All experiments were performed in triplicate, and the results were reported as the mean value. Statistical analysis was performed using one-way ANOVA to test the influence of individual factors on observed properties, with Tukey’s post hoc test used to estimate the differences in the detection of the mean value. Significant levels were considered at *p* ≤ 0.05.

Principal component analysis (PCA) was performed using OriginPro 2023 (OriginLab Corporation, Northampton, MA, USA).

## 3. Results and Discussion

### 3.1. Modeling and Optimization of Pea Pod Extract

#### 3.1.1. Fitting the Model

The extract from pea pod was produced using the principles of green chemistry, via an ultrasound-assisted extraction method, with ethanol serving as the green extraction solvent, and the results are presented in Table 1.

Total phenolic, chlorophyll A, and carotenoid contents showed significant variation depending on the applied extraction conditions (Table 2). The TPC ranged from 2.99 to 5.61 mg GAE/g DW. Chlorophyll A content ranged between 21.49 and 52.04 µg/g DW, while carotenoid content varied from 15.90 to 31.36 µg/g DW (Table 1). The obtained experimental data were successfully fitted to a second-order polynomial model to describe the relationship between the extraction conditions and the responses. The effects of individual factors, their interactions, and the overall adequacy of the models were assessed using analysis of variance (ANOVA) (Table 2). All developed models were statistically significant (*p* < 0.005).

High coefficients of determination (R^2^) were obtained for total polyphenol content (0.91), chlorophyll A content (0.86), and carotenoid content (0.89), indicating good agreement between the experimentally obtained and predicted values. Furthermore, the non-significant lack of fit (*p* > 0.05) confirmed that the models were adequate and reliable for predicting the responses.

#### 3.1.2. Influence of Extraction Conditions on Bioactive Compounds’ Extraction Efficiency and Process Optimization

The obtained results showed that TPC was significantly influenced by the solid-to-solvent ratio (C) and its interaction with the ethanol concentration (AC), as described by the following Equation (2):

(2) TPC = + 4.74 − 0.0675A + 0.9250C + 0.2900AC − 0.4629C^2^,

The positive linear effect of the solid-to-solvent ratio indicates that increasing the solvent volume enhances the extraction of phenolic compounds. This can be explained by mass transfer principles, as a higher concentration gradient between the plant material and the solvent promotes diffusion. However, the negative quadratic effect suggests that, at higher solid-to-solvent ratios within the investigated range, further increases in solvent volume do not result in improvement of the extraction efficiency of phenolic compounds. The interaction between ethanol concentration and the solid-to-solvent ratio had a positive effect on the extraction of polyphenolic compounds, although, within the investigated range, ethanol alone was not statistically significant. This may be explained by the ability of ethanol to break interactions between phenolic compounds and cell wall structural components, such as polysaccharides and proteins [21,22], while an increase in ethanol proportion may also induce protein denaturation, leading to increased viscosity of the plant matrix and consequently hindering the release of phenolic compounds. Thus, a higher solid-to-solvent ratio compensates for viscosity-induced limitations, allowing ethanol to improve polyphenol extraction efficiency.

According to the model proposed for chlorophyll A, the linear and quadratic terms of the solid-to-solvent ratio (C) were the most influential parameters. Also, the interactions between ethanol concentration and time (AB), and between time and the solid-to-solvent ratio (BC), were statistically significant. The equation for the prediction of chlorophyll A content is shown below (Equation (3)):

(3) Chlorophyll A = + 38.12 + 2.28A + 2.58B + 6.70C + 5.83AB + 6.76BC − 7.53C^2^,

Regarding the obtained equation, chlorophyll A content increased with an increase in the solid-to-solvent ratio. However, the negative quadratic effect of the solid-to-solvent ratio revealed that chlorophyll A content increased only up to a certain point, after which the increase of solvent volume was not favorable. This is in agreement with previous studies on chlorophyll extraction from pea pods [23]. Positive interaction effects between extraction time and ethanol concentration, as well as between extraction time and the solid-to-solvent ratio, showed that chlorophyll A content increased when all three investigated factors were at higher levels. This trend can be attributed to the hydrophobic nature of chlorophyll A, indicating that longer extraction times with higher ethanol concentrations and solid-to-solvent ratios improve its extraction.

**Figure 1:**
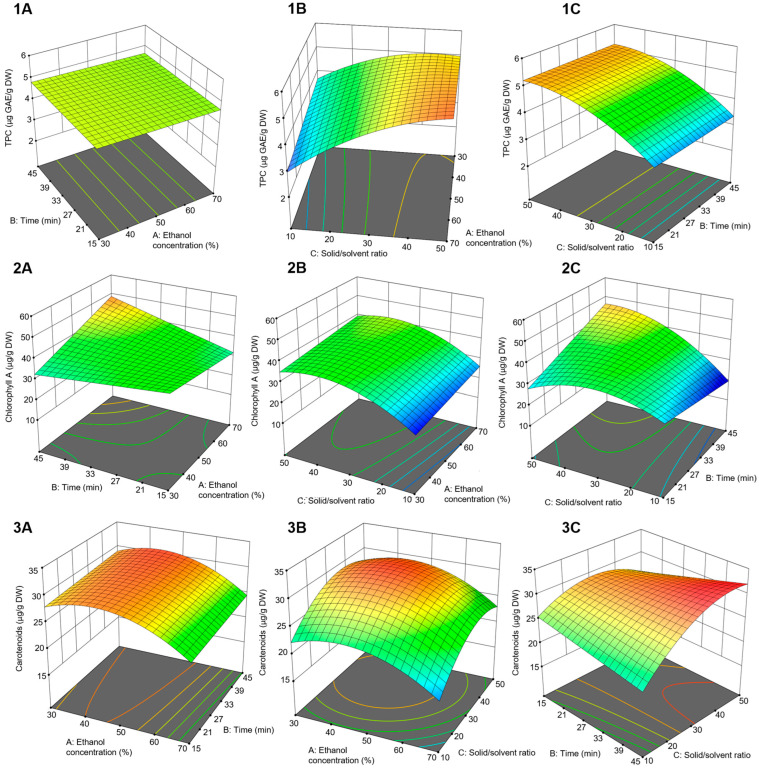
Three-dimensional response surface plots of the yields of (**1A**–**1C**) total phenolic content—TPC, (**2A**–**2C**) chlorophyll A, and (**3A**–**3C**) carotenoids. The color scale represents the magnitude of the response variable, with cooler colors indicating lower values and warmer colors indicating higher value.

Ethanol concentration (A) had the strongest influence on carotenoid yield, followed by solid-to-solvent ratio (C) and interaction between extraction time and the solid-to-solvent ratio (BC). These effects are described in the following Equation (4):

(4) Carotenoids = +29.85 − 2.26A + 0.5669B + 2.68C + 2.50BC − 3.62A^2^ − 3.59C^2^,

The negative linear and quadratic effects of ethanol concentration indicate that the highest carotenoid content was extracted by using 40–50% ethanol (Figure 1). A similar trend was observed during the extraction of carotenoids from carrot pomace, where a high water content (>60%) led to the formation of radicals through ultrasound-induced water dissociation. These reactive species can cause oxidation, which reduces carotenoid extraction efficiency [24]. The positive linear effect of the solid-to-solvent ratio shows that carotenoid content increases with solvent volume. However, the negative quadratic effect indicates that the highest solid-to-solvent ratios do not further improve carotenoid extraction. An exception occurs when both the solid-to-solvent ratio and extraction time are high. This behavior can be explained by the hydrophobic nature of carotenoids, as higher solvent volumes combined with longer extraction times promote their extraction [25].

The objective of this phase of this study was to find the optimal extraction conditions by using the desirability function approach in order to simultaneously maximize the yields of phenolic compounds, chlorophyll A, and carotenoids. The results indicated that the extraction process of the investigated active compounds from pea pods should be performed by using ethanol 58.51% (*v*/*v*) with a solid-to-solvent ratio of 1:48 over 45 min. The desirability of the optimized solution was high, reaching a value of 0.921.

The adequacy of the predictive model was assessed by conducting five replicate experiments under the established optimal conditions, with the results summarized in Table 3. The experimentally obtained mean values agreed with the model predictions, demonstrating the suitability of the developed quadratic models. In addition, the close correlation between the experimental and predicted responses confirms the effective application of response surface methodology for optimizing the extraction of bioactive compounds from pea pods.

The optimal extract for the encapsulation process was produced in higher amounts, under the previously mentioned chosen conditions (ethanol 58.51% (*v*/*v*), a solid-to-solvent ratio of 1:48, over 45 min), at a constant temperature of 50 °C. After the extraction process, ethanol was evaporated at 50 °C to protect the sensitive compounds from thermal degradation. After the evaporation process, a dealcoholized extract was obtained (PPE—pea pod extract), with an alcohol content of 3.24% and a dried matter content of 2.2%.

### 3.2. Characterization of Pea Pod-Dried Extract and Respective Encapsulated Powders

#### 3.2.1. Powder Yield and Moisture Content

Freeze-drying is a widely used technique for producing dry extracts and encapsulated powders, as it preserves heat-sensitive bioactive compounds and allows the preparation of stable powders suitable for storage and further application [26]. The success of the freeze-drying process is commonly evaluated based on powder yield and moisture content, as these parameters directly affect process efficiency, product stability, and economic feasibility [27].

In accordance with these criteria, the encapsulates examined in the present study demonstrated good powder properties, exhibiting high powder yields (with all samples above 90%) and low moisture content, which are essential parameters for further food or pharmaceutical applications (Table 4). The encapsulation yield ranged from 91.5% to 97.7%, with the PPE + FP sample achieving the highest powder yield. No statistically significant differences were observed among the samples with carriers, indicating a robust and efficient freeze-drying process.

The powder yields obtained in this study were markedly higher than those reported in previous studies employing spray drying for pea-based systems. For instance, spray-dried pea matrices have been reported to exhibit recovery yields of approximately 40.7%, primarily due to material adhesion to the drying chamber and the absence of optimized carrier systems [28]. Similarly, pea protein has been used as a carrier for the microencapsulation of beetroot extract via spray drying, yielding powders in the range of 55–72%, depending on formulation and drying temperature [29]. In contrast, the high encapsulation yields achieved in the present work highlight the efficiency of freeze-drying, combined with an appropriately designed formulation, which minimizes material losses and ensures high process recovery. This is the first study reporting the encapsulation of pea pod extracts using the freeze-drying method.

Low moisture content represents a critical quality parameter of dried and encapsulated powders, as it directly influences physicochemical stability, shelf life, and suitability for further processing and application. Elevated residual moisture may accelerate degradation reactions such as oxidation and hydrolysis, promote microbial growth during storage, and negatively affect powder flowability and caking behavior, thereby limiting handling and industrial applicability [20].

In the present study, the moisture content of the freeze-dried powders ranged from 5.48 to 7.63%, indicating an effective lyophilization process capable of substantial water removal while preserving bioactive compounds (Table 3). Variations in moisture content among the samples may be attributed to differences in formulation composition, particularly the presence of protein-rich matrices, which are known to interact with water molecules and may contribute to increased water retention through hydrogen bonding. Among the analyzed samples, pea pod extract encapsulated in pea grain powder, using it as a carrier, exhibited the most favorable moisture content, around 5% which is generally regarded as optimal for preventing microbiological instability in powdered systems. According to the literature, powders with a moisture content below 5% demonstrate enhanced physicochemical and microbiological stability, primarily due to the reduced availability of free water necessary for microbial growth, as well as improved storage performance and an extended shelf life [30]. The moisture content of pure, freeze-dried pea grain powder was 2.9%, indicating good powder and potential carrier properties. The addition of FP during the encapsulation process reduced the moisture content of the PPE.

Additionally, the formulation in which the pea pod extract was encapsulated within a pea-based carrier exhibited a lower moisture content compared to the system containing whey protein as the carrier. This finding suggests improved drying efficiency and potentially enhanced storage stability of the pea-derived encapsulation matrix. The observed differences may be attributed to matrix-specific interactions, including variations in hydrophilicity and protein–polysaccharide interactions, which can influence moisture retention capacity and overall powder stability.

Compared to the spray-dried pea-based powders reported in the literature, which typically exhibit moisture contents below 5%, the values obtained in this study are slightly higher but remain within the range commonly reported for freeze-dried protein-based systems. For instance, freeze-dried emulsions containing a whey protein isolate have shown residual moisture contents ranging from 2.3 to 10.9%, depending on formulation and processing conditions [31]. Similarly, freeze-dried whey protein powders have been reported to contain approximately 8.1% moisture under specific lyophilization parameters [32]. The relatively low moisture content observed in the present work can be attributed to the combined effect of the porous structure generated during freeze drying, which facilitates efficient ice sublimation, and the presence of whey protein and pea-based powders that modulate water distribution within the matrix. Overall, the obtained moisture levels indicate good storage stability and the suitability of the powders for further food or pharmaceutical applications.

#### 3.2.2. Chemical Characterization of Dried Pea Pod Extracts and Their Microencapsulates by Spectrophotometric and HPLC-DAD Analysis

Freeze-dried PPE exhibited moderate levels of bioactive compounds, with a TPC of 16.64 mg/g (Table 5). The pigment profile was characterized by chlorophyll A and B contents of 175.85 μg/g and 253.63 μg/g, respectively, along with a carotenoid content of 104.12 μg/g. The incorporation of PPE in a freeze-dried pea grain powder (PPE + FP) resulted in a decrease in TPC to 10.36 mg/g, while substantially increasing chlorophyll A and B concentrations. The decrease in TPC in PPE + FP can be primarily attributed to a dilution effect caused by the addition of the carrier, as well as possible interactions between phenolic compounds and the pea matrix components (e.g., proteins and polysaccharides), which may reduce their extractability and apparent concentration. The increase in chlorophyll content is likely due to the contribution of endogenous pigments from the pea grain matrix, indicating that the carrier itself acts as an additional source of these compounds.

In contrast, the use of whey protein as a carrier (PPE + WP) provided the most effective retention of bioactive compounds, maintaining TPC at a level comparable to the pure extract (16.41 mg/g) and yielding the highest concentrations of chlorophylls and carotenoids among all the formulations. The superior retention observed in PPE + WP may be attributed to the ability of whey proteins to form a protective matrix around bioactive compounds, reducing their exposure to oxygen and light, and limiting molecular mobility, which in turn decreases degradation during freeze-drying. Despite the generally mild conditions of freeze-drying, partial degradation of phenolic compounds may still occur due to oxidation or structural changes during extraction and drying, which may partly explain the observed differences between formulations.

Comparable trends have been reported in the literature for freeze-dried plant-based systems. Freeze-drying effectively preserves natural pigments, particularly chlorophylls and carotenoids, due to low-temperature processing. For example, freeze-dried protein–spinach particles retained higher chlorophyll levels than spray-dried counterparts [33], and the use of whey protein or maltodextrin as carriers further enhanced pigment stability against pH and light-induced degradation [34].

In addition to chlorophyll preservation, freeze drying has been widely reported to be effective in retaining other heat-sensitive and lipophilic bioactive compounds, including carotenoids and phenolic compounds. Freeze-dried purple carrot products, for instance, demonstrated higher retention of carotenoids and anthocyanins compared to air-dried samples, which was attributed to the low-temperature sublimation process inherent to freeze drying [35]. Moreover, comprehensive reviews indicate that freeze drying generally preserves total phenolic content more efficiently than conventional hot-air or sun drying across a wide range of fruit and vegetable matrices [36].

PPE represents a valuable source of phenolic compounds, predominantly phenolic acids and flavonoids. In the prepared freeze-dried PPE-based encapsulated powders, epicatechin was identified as the most abundant compound, with concentrations ranging from 3.23 to 6.99 μg/g (Table 6). The highest epicatechin content was detected in the PPE + WP sample, followed by the PPE powder without an added carrier, while significantly lower values were observed in the PPE + FP formulation. Gallic acid was also detected in all samples, with contents varying between 1.33 and 1.65 μg/g, with the highest level again recorded in the whey protein-based encapsulate. Catechin, ferulic acid, and sinapinic acid were consistently identified across all formulations, although a general decrease in their concentrations was observed in the samples containing FP as a carrier (Table 6).

In contrast, coumaric acid exhibited a different trend, with its content being approximately twofold higher in the PPE + FP sample compared to the other encapsulated powders. This observation suggests that the FP-based matrix may favor the retention of certain phenolic acids during freeze drying, possibly due to specific interactions between native pea components and coumaric acid. Coumaric acid is a naturally occurring phenolic compound widely distributed in fruits, vegetables, and cereals, and is known for its antioxidant, anti-inflammatory, cardioprotective, and antimicrobial properties [37,38]. Kaempferol was detected in relatively low amounts in all samples, with slightly higher values observed in the PPE + FP formulation.

The variations observed in the phenolic profiles among the encapsulated samples indicate that the carrier composition likely played an important role in the retention of individual bioactive compounds during the freeze-drying process. Protein-based carriers, particularly whey protein, may provide a more protective microenvironment for phenolic compounds through protein–polyphenol interactions, such as hydrogen bonding and hydrophobic interactions, which have been reported to enhance phenolic stability during drying processes. Similar trends have been described in previous studies, where improved retention of flavonoids and phenolic acids was observed in protein-rich encapsulation systems, especially under mild drying conditions such as freeze drying [39].

Conversely, the generally lower levels of most phenolic compounds observed in FP-containing samples may be associated with the more complex composition of freeze-dried pea material, which comprises proteins, carbohydrates, and other endogenous components that can compete with phenolic compounds for interaction sites within the matrix [40]. Interestingly, the increased content of coumaric acid in the PPE + FP formulation suggests that this compound may exhibit a higher affinity or improved stability within the pea-based matrix, a phenomenon that has also been reported previously [41]. Overall, these findings emphasize that the effectiveness of encapsulation depends not only on the applied drying technique but also on the physicochemical compatibility between the carrier material and specific phenolic compounds.

### 3.3. Physical Characterization of Powdered Pea Pod Extract and Its Microencapsulates

#### 3.3.1. Fourier Transform Infrared Spectroscopy (FTIR)

**Figure 2:**
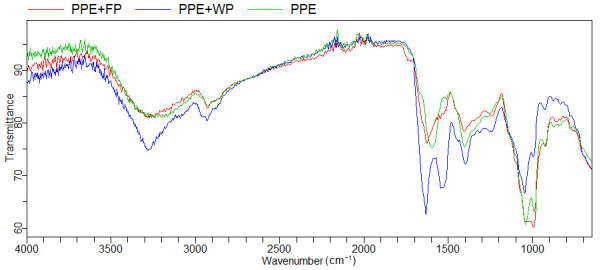
FTIR analysis of pea pod extract (PPE), and respective encapsulated powders of PPE with pea grain powder (FP) and whey protein (WP).

FTIR analysis provided information about encapsulated guest molecules of the pea pod extract and its encapsulated forms in selected biopolymers (Figure 2). The spectra exhibited a broad and intense absorption band around 3250 cm^−1^, originating from the O–H stretching vibrations of hydroxyl groups. This band is typical of phenolic compounds and polysaccharides and is commonly reported as a key marker of polyphenol presence in plant extracts [14,20]. Similar broad O–H bands in the range 3200–3400 cm^−1^ were reported for phenolic-rich extracts of legumes and other plant materials, confirming that this region is associated with hydrogen-bonded hydroxyl groups of flavonoids, phenolic acids, and other polyphenolic compounds [42]. The persistence of this band after freeze-drying encapsulation indicates that the phenolic structures were preserved, which is consistent with reports showing that freeze-drying effectively maintains the chemical integrity of phenolics compared to thermal drying methods [12,36,43].

In the region 1000–1100 cm^−1^, corresponding to C–O and C–O–C stretching vibrations, all samples displayed characteristic carbohydrate-related bands. These bands were more pronounced in PPE and PPE + FP, confirming the dominance of a polysaccharide matrix and suggesting that phenolic structures remained incorporated without structural degradation. Similar bands were observed in the FTIR spectra of encapsulated polyphenols in carbohydrate-based matrices, such as maltodextrin, gum Arabic, and starch, where peaks near 1020–1080 cm^−1^ were associated with C–O–C and C–O stretching of glycosidic bonds [44,45]. The presence of these bands in the pea-based powder and in the encapsulated system with a pea-based carrier confirms the dominance of a polysaccharide-rich matrix and indicates that aromatic phenolic structures from the pea pod extract remained intact. For the whey protein-based system (PPE + WP), characteristic Amide I (~1650 cm^−1^) and Amide II (~1540 cm^−1^) bands were observed. These bands are typical for protein-based matrices and are widely reported in the FTIR studies of protein–polyphenol systems. Hasni et al. (2011) [46] and Rawel et al. (2005) [47] reported similar Amide I and II bands when milk proteins interacted with phenolic compounds, and small shifts in these bands were associated with hydrogen bonding and hydrophobic interactions between phenolics and protein functional groups. Slight changes in band intensity and position compared to the pure carrier indicate weak interactions between phenolic compounds and protein functional groups, likely through hydrogen bonding and hydrophobic interactions, as commonly reported for protein–polyphenol systems.

In contrast, the encapsulated system using pea-based powder as an innovative carrier showed spectra dominated by polysaccharide features, with strong bands in the 1000–1150 cm^−1^ region and a broad O–H band around 3250 cm^−1^. The slight broadening and intensity changes of the O–H band in the pea-based encapsulated system indicate the formation of hydrogen bonds between phenolic hydroxyl groups and polysaccharide chains, which is a mechanism frequently described for polyphenol–carbohydrate interactions [48].

Importantly, no new peaks or disappearances of characteristic bands were observed in the encapsulated samples. The spectra represent a superposition of extract and carrier signals, indicating that encapsulation occurred through physical incorporation rather than chemical modification. This behavior supports the formation of non-covalent interactions, which is typical for the encapsulation of polyphenols in biopolymer matrices [48,49].

A comparison of the two carriers reveals distinct interaction mechanisms: whey protein primarily promotes phenolic–protein interactions, while the pea-based carrier favors phenolic–polysaccharide interactions, as evidenced by changes in the O–H and carbohydrate regions. These results confirm successful encapsulation in both systems, with differences in interaction type depending on the carrier matrix.

#### 3.3.2. Differential Scanning Calorimetry (DSC) Analysis

**Figure 3:**
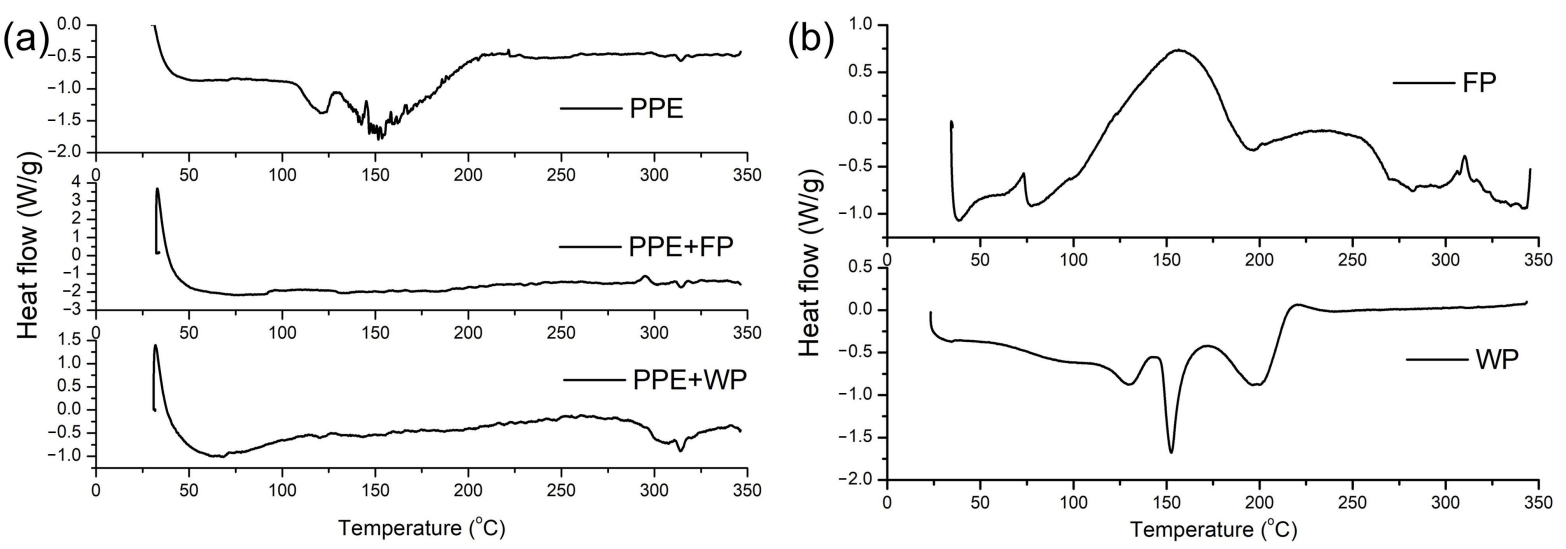
DSC thermograms of (**a**) pea pod extract (PPE) encapsulated in pea grain powder (FP) and whey protein (WP); (**b**) respective carriers, pea grain powder (FP) and whey protein (WP).

To evaluate the thermal stability of pea pod extract (PPE) and its encapsulated forms, differential scanning calorimetry (DSC) analysis was performed. DSC thermograms were recorded for the dried pea pod extract, PPE encapsulated powders (Figure 3a), and encapsulation carriers used in this study (Figure 3b), freeze-dried pea grain powder as a potential innovative carrier, and whey protein as a conventionally used carrier (Figure 3). Together with FTIR analysis, DSC enables the monitoring of interactions between encapsulated phenolic compounds, natural pigments, and carrier matrices, as well as their influence on thermal behavior.

According to the obtained DSC data, encapsulated PPE systems were stable in a temperature range of approximately 127–253 °C, which is highly relevant for food processing operations such as baking, roasting, extrusion, and drying, where ingredients are often exposed to elevated temperatures during processing (Table 7). This also supports the potential use of these encapsulates in pharmaceutical formulations, where thermal stability is essential for maintaining bioactivity during manufacturing and storage. The literature data indicate that encapsulated plant extracts intended for functional foods or nutraceuticals are generally considered technologically suitable if they remain stable up to at least 120–150 °C [50,51]. Therefore, the stability of PPE encapsulated within this temperature range confirms its high applicability.

The addition of freeze-dried pea grain powder (FP) to PPE clearly increased the degradation temperature of the extract compared to pure dried PPE, demonstrating that encapsulation significantly improves thermal stability. Similar shifts toward higher degradation or transition temperatures have been reported for phenolic compounds encapsulated in starch-, gum-, or legume-based matrices, confirming the protective effect of plant-derived carriers [52].

DSC analysis also showed that the degradation peak temperatures of pea-based encapsulates reached similar temperature ranges and even higher enthalpy changes compared to whey-protein-based systems. Higher enthalpy values indicate that more energy is required to induce thermal transitions, reflecting stronger structural organization and the stability of the carrier matrix. Comparable results have been reported for plant-based carriers, which often exhibit higher enthalpy changes than purely protein-based systems due to combined polysaccharide–protein network formation [50,51].

Based on the obtained thermograms, all examined encapsulates can be considered thermally stable within a temperature range relevant for food processing and potential pharmaceutical applications. In the present study, lyophilized carriers clearly improved the thermal resistance of PPE, probably by forming a protective matrix around extract droplets or particles. This matrix reduces molecular mobility and delays thermal degradation, a mechanism widely described for protein- and polysaccharide-based encapsulation systems [50,53].

In agreement with the recent literature, DSC analysis confirmed that encapsulation significantly enhances the thermal stability of plant phenolics [54,55]. Particular attention was given to the natural-based carrier, freeze-dried pea grain powder, as a potential biopolymer. Pea grain powder exhibited the highest thermal stability, with the highest degradation peak temperature among all the analyzed samples. The innovative pea-based carrier demonstrated particularly strong thermal resistance, comparable to or even exceeding that of conventional whey protein carriers, which are widely reported as effective thermal protectants of polyphenols [56]. The whey protein carrier *β*-lactoglobulin (a major whey protein) can form complexes with epigallocatechin gallate that exhibit improved stability and resistance to degradation relative to unencapsulated polyphenols and can enhance the stability of the mentioned polyphenolic compounds through protein–polyphenol interactions [57]. Studies on peas and other legumes show that legume-derived powders and protein–polysaccharide matrices naturally possess high thermal stability due to their dense macromolecular structure and strong intermolecular interactions, making them suitable for technological and pharmaceutical applications [58]. Moreover, legume proteins have been reviewed as effective carriers for the encapsulation of unstable bioactive compounds due to their robust structural features and ability to form stable networks with polysaccharides [59].

The observed increase in degradation temperature and enthalpy after encapsulation indicates that both carriers form an effective protective matrix, delaying thermal degradation of bioactive compounds [54,56]. However, the pea-based system combines this technological advantage with the additional benefit of being plant-derived, sustainable, and compatible with current trends toward plant-based functional and pharmaceutical products [59]. These findings therefore strongly support the use of freeze-dried pea grain powder as an innovative, natural, and thermally robust carrier for pea pod extract.

#### 3.3.3. Particle Size Distribution

**Figure 4:**
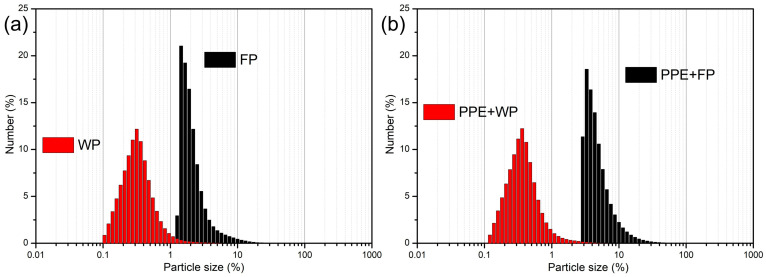
Particle size distribution of (**a**) pea pod extract (PPE) encapsulated in whey protein (WP) and pea grain powder (FP); (**b**) respective carriers, whey protein (WP) and pea grain powder (FP).

The particle size distribution of the freeze-dried encapsulated powders and their respective carriers was determined by laser diffraction (Figure 4, Table 8), presented by modal distribution. The obtained particles were within the microparticle range, with diameters spanning from 0.176 µm (d_10_) to 8.01 µm (d_90_), and median values (d_50_) between 0.33 and 3.93 µm [14,44,45]. Such size ranges are typical for freeze-dried polyphenol-rich systems and are considered suitable for effective encapsulation and controlled release of bioactive compounds. Notably, the particle size distribution of the pure PPE could not be determined due to melting during measurement, emphasizing the essential role of carrier materials in stabilizing and structuring the extract.

Clear differences were observed between the two carrier systems. The whey protein-based encapsulates (PPE + WP) exhibited significantly smaller particle sizes (d_50_ ≈ 0.33 µm), indicating the formation of compact matrices [49,50]. A smaller particle size is generally associated with higher surface area, which may facilitate a faster release of encapsulated compounds. However, the relatively broader size distribution observed in these systems suggests structural heterogeneity, which may result in less controlled release behavior.

In contrast, the pea-based encapsulates (PPE + FP) showed larger particle sizes but narrower size distributions (lower span values), indicating a more homogeneous particle population. Such characteristics are often associated with more uniform encapsulation and, potentially, a more controlled and sustained release of bioactive compounds. This behavior can be attributed to the physicochemical properties of polysaccharide-rich carriers, which tend to form more hydrated and swollen matrices during freeze-drying, resulting in larger but structurally consistent particles [44,60].

The observed differences highlight distinct encapsulation mechanisms: protein-based systems favor the formation of smaller particles with a faster release potential, while polysaccharide-based systems promote larger, more uniform particles that may support a prolonged release. This is in agreement with the literature data on protein- and carbohydrate-based encapsulation systems, where matrix composition directly influences particle formation and release kinetics. Generally, the WP addition in the PPE resulted in particle sizes similar to those of pure WP, suggesting that the incorporation of the extract did not markedly disrupt the protein matrix. This behavior has also been reported in studies where a polyphenol addition did not significantly change the size of protein-based microcapsules, because phenolics are mostly entrapped within pre-formed protein networks [46]. Although the addition of FP as a carrier generated a particle size in PPE + FP encapsulates that is higher compared to PPE + WP, FP, as a potential innovative carrier, indicates good particle uniformity and a potentially longer release of bioactive compounds, which is typical of particles with a higher diameter. Similar findings were reported for starch and fiber-based carriers, where larger particles were obtained but with relatively narrow size distributions due to uniform swelling of the polysaccharide matrix [45,60]. Distribution width and dispersion homogeneity were assessed through Span and Uniformity parameters. Whey protein-based systems (PPE + WP and WP) exhibited significantly smaller median particle diameters (d_50_ ≈ 0.33 µm) compared to pea grain powder-based formulations. However, their higher relative distribution width (d_90_/d_10_ ≈ 3.67) suggests a broader particle size distribution. In contrast, pea-based systems (PPE + FP and FP) showed lower d_90_/d_10_ ratios (≈2.80 and ≈2.54), indicating a comparatively narrower size distribution despite larger absolute particle diameters. Importantly, the narrower distributions observed in pea-based matrices may indicate more uniform carrier–core interactions and potentially more consistent encapsulation efficiency. In contrast, whey protein systems, although generating finer particles, may exhibit greater heterogeneity due to protein aggregation phenomena during drying.

The narrower particle size distribution and lower moisture content observed for the pea-based system suggest improved physical stability, a reduced agglomeration tendency, and enhanced structural integrity, which are critical parameters for storage and application performance.

The presented results demonstrate that both carrier systems enable the formation of microparticles suitable for the encapsulation of pea pod extract, while differences in particle size distribution indicate that the choice of carrier can be used to tailor the release behavior and functional performance of the final formulation [43,49].

### 3.4. PCA Analysis

**Figure 5:**
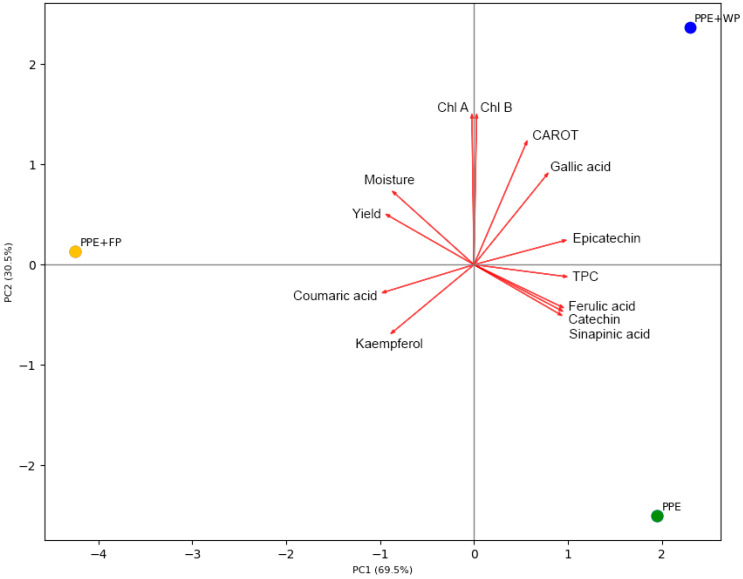
Principal component analysis biplot of PC1 versus PC2. The markers represent the scores of the samples: (1) PPE, the pure pea pod extract; (2) PPE + FP, combined with freeze-dried pea grain powder; (3) PPE + WP, combined with whey protein, on the PC1 and PC2. The red arrows indicate the PC loading vectors.

To visually analyze the data describing pea pod encapsulates, a PCA analysis was performed. The PCA biplot presented in Figure 5 revealed that the variables could be reduced to two principal components, explaining 100% of the total variance, with PC1 and PC2 accounting for 69.5% and 30.5% of the variability, respectively.

PC1 was primarily influenced by TPC, as well as the levels of individual phenolic compounds, including epicatechin, catechin, ferulic acid, sinapic acid, and gallic acid, therefore indicating differences in phenolic profiles among the samples. The similar lengths of the corresponding loading vectors suggested comparable contributions of these variables to PC1. In contrast, the very small angles between the catechin, ferulic acid, and sinapic acid vectors indicated a strong positive correlation among these compounds. In contrast, kaempferol and coumaric acid exhibited negative loadings on PC1. PC2 was primarily influenced by photosynthetic pigments, with chlorophyll A, chlorophyll B, and carotenoids exhibiting strong positive effects, thereby distinguishing samples based on their pigment content.

The score plot revealed a clear separation among the analyzed pea pod samples. Higher yield as well as favorable moisture content were closely associated with the sample prepared with the freeze-dried pea grain powder (PPE + FP). However, the yield of individual phenolic compounds showed a specific distribution related to the different prepared pea pod samples. PPE + FP was positioned on the negative side of PC1, corresponding to the higher relative contributions of kaempferol and coumaric acid, whereas the pure pea pod sample (PPE) exhibited positive correlations with PC1, together with the TPC, and levels of catechin, ferulic acid, and sinapic acid. On the other hand, the sample prepared with whey protein (PPE + WP) was associated with a higher content of epicatechin and gallic acid. Both carrier-containing samples, PPE + FP and PPE + WP, were positioned closer to pigment-related variables, including chlorophyll A, chlorophyll B, and carotenoids, indicating a higher association with pigment content compared to PPE. Overall, the PCA demonstrated that PC1 primarily reflects variation in phenolic composition, while PC2 describes differences in pigment content, collectively highlighting distinct compositional profiles among the samples.

## 4. Conclusions

This study presents a sustainable and plant-oriented approach to the valorization of pea pod residues, confirming their potential as a rich source of polyphenols and natural pigments. By applying ultrasound-assisted green extraction and response surface methodology, the extraction conditions were successfully optimized to maximize the recovery of target bioactive compounds under environmentally friendly conditions, reinforcing the relevance of pea pods as an underutilized plant-derived resource. The optimized extract was effectively stabilized through freeze-drying, yielding powders with high process efficiency and favorable technological properties. A comparative evaluation of the carrier systems demonstrated that whey protein ensured higher retention of total phenolics and pigments, whereas freeze-dried pea grain powder enabled the development of an innovative, fully plant-based encapsulation system. This “pea within pea” strategy represents a novel concept in legume valorization, integrating pea pod bioactives with a pea-derived carrier and supporting complete crop utilization. Physicochemical analyses (FTIR, DSC, and particle size distribution) confirmed that encapsulation occurred through physical interactions without structural degradation of bioactive compounds and significantly enhanced the thermal stability of the extract. Differences observed in phenolic profiles among formulations highlight the role of carrier composition in modulating the retention of individual compounds. Overall, the findings support the use of pea pod residues as valuable plant material and demonstrate that pea-based carriers can serve as sustainable alternatives to conventional protein systems. This work contributes to the expanding field of plant resource valorization and circular bioeconomy, while highlighting the potential of pea pod residues, at the laboratory scale, as a promising source for the future development of functional food ingredients and plant-based nutraceutical formulations. Future research will focus on evaluating economic feasibility, scalability, and potential practical applications.

## Figures and Tables

**Table 1:** Box–Behnken design for three factors with actual and coded values, and experimentally obtained responses.

	Factors			Responses		
Sample	A: Ethanol Concentration (%)	B: Time (min)	C: Solid/Solvent Ratio	TPC (mg GAE/g DW)	ChlA (µg/g DW)	Carotenoids (µg/g DW)
1	30 (−1)	15 (−1)	30 (0)	4.82	37.82	27.65
2	70 (+1)	15 (−1)	30 (0)	4.29	37.65	22.01
3	30 (−1)	45 (+1)	30 (0)	4.68	28.88	26.77
4	70 (+1)	45 (+1)	30 (0)	4.73	52.04	26.91
5	30 (−1)	30 (0)	10 (−1)	3.53	25.10	23.20
6	70 (+1)	30 (0)	10 (−1)	2.92	21.55	15.90
7	30 (−1)	30 (0)	50 (+1)	5.06	37.84	29.15
8	70 (+1)	30 (0)	50 (+1)	5.61	36.63	23.91
9	50 (0)	15 (−1)	10 (−1)	3.42	27.41	26.37
10	50 (0)	45 (+1)	10 (−1)	3.55	21.49	21.63
11	50 (0)	15 (−1)	50 (+1)	4.63	26.77	25.10
12	50 (0)	45 (+1)	50 (+1)	5.52	47.90	30.35
13	50 (0)	30 (0)	30 (0)	5	38.09	31.36
14	50 (0)	30 (0)	30 (0)	4.73	31.92	30.30
15	50 (0)	30 (0)	30 (0)	4.95	40.45	29.48

**Table 2:** Estimated regression coefficients and analysis of the variance of the fitted second-order polynomial models.

Source	df ^a^	SS	MS	F-Value	*p*-Value
**TPC**					
Model	4	8.02	2.00	24.19	<0.0001
A-Ethanol concentration	1	0.0365	0.0365	0.4400	0.5221
C-Solid/solvent ratio	1	6.84	6.84	82.62	<0.0001
AC	1	0.3364	0.3364	4.06	0.0716
C^2^	1	0.7998	0.7998	9.65	0.0111
Error	10	0.8285	0.0828		
Lack of fit	8	0.7872	0.0984	4.77	0.1275
Pure Error	2	0.0413	0.0206		
Total	14	8.85			
R^2^	0.9063				
Adjusted R^2^	0.8689				
**Chlorophyll A**					
Model	6	984.60	164.10	8.28	0.0044
A-Ethanol concentration	1	41.50	41.50	2.09	0.1860
B-Time	1	53.41	53.41	2.69	0.1394
C-Solid/solvent ratio	1	358.97	358.97	18.10	0.0028
AB	1	135.92	135.92	6.85	0.0307
BC	1	182.91	182.91	9.22	0.0161
C^2^	1	211.89	211.89	10.69	0.0114
Error	8	158.63	19.83		
Lack of fit	6	119.88	19.98	1.03	0.5685
Pure Error	2	38.76	19.38		
Total	14	1143.23			
R^2^	0.8612				
Adjusted R^2^	0.7572				
**Carotenoids**					
Model	6	215.58	35.93	10.66	0.0019
A-Ethanol concentration	1	40.72	40.72	12.08	0.0084
B-Time	1	2.57	2.57	0.7625	0.4080
C-Solid/solvent ratio	1	57.30	57.30	16.99	0.0033
BC	1	24.99	24.99	7.41	0.0262
A^2^	1	48.58	48.58	14.41	0.0053
C^2^	1	47.85	47.85	14.19	0.0055
Error	8	26.97	3.37		
Lack of fit	6	25.20	4.20	4.74	0.1843
Pure Error	2	1.77	0.8855		
Total	14	242.55			
R^2^	0.8888				
Adjusted R^2^	0.8054				

^a^ The “Degrees of Freedom (df)” column also includes the coefficient of determination (R^2^). Abbreviations: SS—sum of squares; MS—mean square.

**Table 3:** Total phenolic, chlorophyll A, and carotenoid content in optimal pea pod extract (optimal PPE), dealcoholized pea pod extract (dealcoholized PPE), and freeze-dried pea (FP).

Sample	TPC (mg GAE/g DW)	Chl A (µg/g DW)	CAROT (µg/g DW)
Optimal PPE	5.36 ± 0.29	46.88 ± 3.11	29.22 ± 2.81
CI 95%	4.96–5.60	42.32–58.01	27.36–33.75
TI 95%	3.84–6.72	24.21–76.12	19.89–41.22
Dealcoholized PPE	6.43 ± 0.33	52.38 ± 3.21	25.38 ± 2.11
FP	22.36 ± 1.25	3092.08 ± 33.69	11.06 ± 2.25

Abbreviations: TI—Tolerance Interval; CI—Confidence Interval.

**Table 4:** Technological properties of freeze-dried powders.

Samples	Yield (%)	Moisture (%)
PPE	91.5 ^b^	7.63 ^a^
PPE + WP	93.35 ^ab^	6.67 ^b^
PPE + FP	97.72 ^a^	5.48 ^c^

Different letters indicate statistically significant differences within the same column according to Tukey’s test (*p* < 0.05).

**Table 5:** Total phenolic content, green pigments, and carotenoid contents in dried pea pod extracts and respective encapsulated powders.

Sample	TPC (mg GAE/g DW)	Chl A (µg/g DW)	Chl B (µg/g DW)	CAROT (µg/g DW)
PPE	16.64 ^a^*	175.85 ^c^	253.63 ^c^	104.12 ^b^
PPE + WP	16.41 ^a^	2998.85 ^a^	4674.62 ^a^	250.53 ^a^
PPE + FP	10.40 ^b^	1758.40 ^b^	2539.86 ^b^	98.33 ^b^

* Different letters indicate statistically significant differences within the same column according to Tukey’s test (*p* < 0.05).

**Table 6:** Results of HPLC analysis of pea pod extract-based encapsulated powders.

Individual Compounds	PPE (μg/g)	PPE + WP (μg/g)	PPE + FP (μg/g)
Gallic acid	1.44 ± 0.04 ^b^*	1.65 ± 0.04 ^a^	1.33 ± 0.08 ^c^
Epicatechin	6.16 ± 0.20 ^b^	6.99 ± 0.20 ^a^	3.23 ± 0.09 ^c^
Catechin	1.63 ± 0.03 ^a^	1.48 ± 0.03 ^b^	1.08 ± 0.03 ^c^
Coumaric acid	0.87 ± 0.02 ^b^	0.69 ± 0.02 ^a^	1.43 ± 0.01 ^c^
Ferulic acid	0.36 ± 0.02 ^a^	0.31 ± 0.02 ^b^	0.16 ± 0.01 ^c^
Sinapinic acid	1.63 ± 0.03 ^a^	1.39 ± 0.02 ^b^	0.83 ± 0.02 ^c^
Kaempferol	0.04 ± 0.00 ^b^	0.03 ± 0.00 ^c^	0.05 ± 0.00 ^a^

* Different letters indicate statistically significant differences within the same row according to Tukey’s test (*p* < 0.05).

**Table 7:** The transition temperatures and enthalpy changes of pea pod extract, encapsulates, and their respective carriers.

Samples	T1	T2	T3	T4	∆H1	∆H2	∆H3	∆H4
FP	212.88 ± 36.07 ^a^	0.00 ± 0.00 ^b^	0.00 ± 0.00 ^b^	0.00 ± 0.00 ^b^	106.97 ± 18.90 ^a^	0.00 ± 0.00 ^b^	0.00 ± 0.00 ^b^	0.00 ± 0.00 ^b^
PPE	120.38 ± 15.31 ^b^	151.68 ± 15.36 ^a^	0.00 ± 0.00 ^b^	0.00 ± 0.00 ^b^	27.46 ± 2.52 ^c^	174.85 ± 21.16 ^a^	0.00 ± 0.00 ^b^	0.00 ± 0.00 ^b^
PPE + FP	183.78 ± 29.53 ^ab^	0.00 ± 0.00 ^b^	0.00 ± 0.00 ^b^	0.00 ± 0.00 ^b^	73.12 ± 10.83 ^b^	0.00 ± 0.00 ^b^	0.00 ± 0.00 ^b^	0.21 ± 0.03 ^b^
PPE + WP	197.06 ± 32.03 ^a^	0.00 ± 0.00 ^b^	0.00 ± 0.00 ^b^	0.00 ± 0.00 ^b^	60.74 ± 7.57 ^b^	0.00 ± 0.00 ^b^	0.00 ± 0.00 ^b^	0.00 ± 0.00 ^b^
WP	92.11 ± 12.31 ^c^	129.62 ± 14.23 ^a^	152.58 ± 13.52 ^a^	200.76 ± 29.74 ^a^	6.66 ± 0.99 ^c^	25.04 ± 2.89 ^b^	54.66 ± 8.16 ^a^	104.28 ± 8.72 ^a^

Abbreviations: T—temperature transition; ∆H—enthalpy change; FP—freeze-dried pea grain powder; PPE—pea pod extract; WP—whey protein. Different letters indicate statistically significant differences within the same row according to Tukey’s test (*p* < 0.05).

**Table 8:** The particle size of pea pod extract microencapsulates, and the carriers used for microencapsulation.

Samples	d10 *	d50 **	d90	Span ***	D (4.3)	D (3.2)	Uniformity
PPE + FP	2.86 ± 0.45 ^a^	3.93 ± 0.42 ^a^	8.01 ± 0.79 ^a^	1.31 ± 0.14 ^a^	83.22 ± 10.40 ^a^	25.51 ± 2.26 ^a^	0.47 ± 0.04 ^a^
PPE + WP	0.18 ± 0.01 ^c^	0.33 ± 0.02 ^c^	0.66 ± 0.10 ^c^	1.46 ± 0.23 ^a^	11.09 ± 1.84 ^bc^	4.19 ± 0.37 ^c^	0.58 ± 0.07 ^a^
FP	1.33 ± 0.07 ^b^	1.75 ± 0.25 ^b^	3.38 ± 0.27 ^b^	1.18 ± 0.08 ^a^	24.24 ± 2.87 ^b^	10.44 ± 1.21 ^b^	0.45 ± 0.07 ^a^
WP	0.18 ± 0.02 ^c^	0.33 ± 0.05 ^c^	0.66 ± 0.07 ^c^	1.14 ± 0.18 ^a^	8.13 ± 0.52 ^c^	3.50 ± 0.49 ^c^	0.57 ± 0.04 ^a^

* d_10_, d_50_, d_90_ signify the sizes where 10%, 50%, and 90% of the microparticles are smaller than the remaining particles, ** mean diameter, *** calculated as (d_90_ − d_10_)/d_50_. Abbreviations: PPE—pea pod extract; FP—freeze-dried pea grain powder; WP—whey protein. Different letters indicate statistically significant differences within the same row according to Tukey’s test (*p* < 0.05).

## Data Availability

The original contributions presented in this study are included in the article. Further inquiries can be directed to the corresponding author.
